# Increasing Expiratory Hydrogen in Lactose Intolerance Is Associated with Additional Food Intolerance/Malabsorption

**DOI:** 10.3390/nu12123690

**Published:** 2020-11-30

**Authors:** Wolfgang J. Schnedl, Nathalie Meier-Allard, Sonja Lackner, Dietmar Enko, Harald Mangge, Sandra J. Holasek

**Affiliations:** 1 General Internal Medicine Practice, Theodor Körnerstrasse 19b, A-8600 Bruck, Austria; 2 Division of Immunology and Pathophysiology, Otto Loewi Research Center, Medical University of Graz, Heinrichstrasse 31a, A-8010 Graz, Austria; nathalie.allard@medunigraz.at (N.M.-A.); sonja.lackner@medunigraz.at (S.L.); sandra.holasek@medunigraz.at (S.J.H.); 3 Clinical Institute of Medical and Chemical Laboratory Diagnosis, Medical University of Graz, Auenbruggerplatz 30, A-8036 Graz, Austria; dietmar.enko@gmx.at (D.E.); harald.mangge@medunigraz.at (H.M.)

**Keywords:** lactose intolerance, hydrogen breath test, histamine intolerance, diamine oxidase, fructose malabsorption

## Abstract

Single and/or combined food intolerance/malabsorption may cause nonspecific, functional gastrointestinal (GI) complaints. In lactose-intolerant patients we evaluated the influence of additional food intolerance/malabsorption with hydrogen (H_2_) breath tests. In a retrospective analysis of charts from 279 lactose-intolerant patients, we found 128 patients with only lactose intolerance (LIT). Then, we identified 106 LIT patients with additional histamine intolerance (HIT). Additionally, 45 LIT and HIT patients also had fructose malabsorption (FM). A hydrogen (H_2_) breath test was performed to evaluate LIT and FM. A serum diamine oxidase value of <10 U/mL and a response to a histamine-reduced diet was used to identify HIT. Using pairwise comparison with the Kruskal–Wallis test to associate the area under the curve (AUC) of LIT patients and, LIT with HIT, to LIT with HIT and FM it was found, that the exhaled hydrogen values were significantly higher in patients with two-fold and triple combined food intolerance/malabsorption (*p* < 0.004 and *p* < 0.001, respectively). Within the pool of 170 LIT patients with >20 ppm increase of expiratory H_2_ from baseline, there were 74 LIT-only patients, 60 LIT with HIT patients, and 36 LIT patients with additional HIT and FM. With the Kruskal–Wallis test AUCs demonstrated a significant difference between all three groups (*p* = 0.024). In patients with LIT, the presence of additional food intolerance/malabsorption, significantly increases expiratory H_2_ values. We demonstrate evidence, which may suggest HIT to embody an own GI disorder as food intolerance/malabsorption.

## 1. Introduction

Functional, nonspecific, non-allergic gastrointestinal complaints (FNNGIC) and gastrointestinal (GI) disorders, including irritable bowel syndrome (IBS) and IBS-like syndromes, are widespread and a main reason for consultations in primary care [1]. These functional abdominal, symptom-based, syndromes have a symptom profile comparable to food intolerance/malabsorption including fructose malabsorption (FM), histamine intolerance (HIT), and lactose intolerance (LIT) [2]. Combined appearance of these is increasingly reported and occurs in more than 30% of patients with FNNGIC and food intolerance/malabsorption [3,4]. For the clinical diagnosis of LIT and FM proved hydrogen (H_2_) breath tests useful. The diagnosis of HIT is challenging but the diagnosis of HIT may be supported with the measurement of the enzyme diamine oxidase (DAO) in serum. In HIT a disproportionate amount of histamine in the body is thought to result from the consumption of food with high histamine content, and a reduced ability of mainly DAO to digest histamine [5]. In this study we evaluated 279 lactose-intolerant patients for additional food intolerance/malabsorption. Increasing expiratory H_2_ values during H_2_ lactose breath tests are demonstrated with the presence of additional food intolerance/malabsorption. Moreover, we demonstrate evidence, that may suggest HIT to embody an own food intolerance/malabsorption. We recommend that each patient with FNNGIC needs triggers of food intolerance/malabsorption, including HIT, evaluated. If detected, then a registered and experienced dietician is essential to design an individual diet to achieve long-term symptom reduction.

## 2. Methods

In a retrospective analysis of charts from lactose-intolerant patients we identified 279 consecutive lactose-intolerant patients who were evaluated for additional food intolerance/malabsorption, including FM and HIT. All of the patients presented nonspecific, non-allergic functional GI complaints. Main symptoms indicated were abdominal pain, flatulence, loose stools, diarrhea, and postprandial fullness. Patients were divided into three diagnostic groups according to results of their H_2_ breath tests: (1) diagnosis of LIT with increase of H_2_ >20 parts per million (ppm) only, but blood glucose (BG) increase >20 mg/dL; (2) diagnosis of LIT with increase of H_2_ >20 ppm combined with BG increase <20 mg/dL; (3) diagnosis of LIT with BG increase <20 mg/dL only, and increase of expiratory H_2_ <20 ppm.

The H_2_ breath test (Gastrolyzer, Bedfont Scientific Inc., Kent, England) was used to evaluate LIT and FM. The lactose H_2_ test was performed with 200 mL water, containing 50 g of lactose. The end-expiratory exhalation of H_2_ was measured every 30 min for a period of 120 min. Blood glucose was determined with a glucose oxidase method, fasting and measuring every 60 min for a period of 120 min. If the expiratory H_2_ value increased from the baseline for more than 20 parts per million (ppm), and/or BG increased less than 20 mg/dL, then a LIT was diagnosed. For FM, the H_2_ breath test was performed with a drink containing 25 g of fructose in 200 mL water. If the H_2_ value increased from the baseline for more than 20 ppm, then FM was diagnosed.

Before the breath tests no antibiotics or laxatives were allowed, and no colonoscopy was performed within 4 weeks. In the morning, at the first presentation, blood drawings were performed after overnight fasting (>12 h) and the H_2_ lactose breath testing was started without having smoked, chewed gum, or performed vigorous exercise for at least 4 h.

Generally, in our outpatient clinic, we include serum diamine oxidase (DAO) determination in the evaluation of patients presenting with recurring, functional, nonspecific abdominal complaints. The DAO in serum was determined through the radio extraction assay, DAO Rea 100 (Sciotec Diagnostic Technologies, Tulln, Austria). A registered dietician was consulted to introduce and observe an individual food intolerance/malabsorption-reduction and/or -elimination diet. A thorough anamnesis, concerning abdominal complaints, and a timely relation to the ingestion of food or drinks, including pharmaceutical treatments, that might influence FNNGIC, was performed.

In all evaluated patients, either histologic evaluation of gastric mucosa or an enzyme-linked IgA immunosorbent assay (ELISA, Serion, Würzburg, Germany) were used to detect *Helicobacter pylori* infection. For screening of celiac disease, antibodies against tissue transglutaminase were determined with anti-tTG IgA ELISA (Euro Diagnostica AB, Malmö, Sweden). Patients with diabetes mellitus were not included in this evaluation.

The study follows the ethical guidelines of the Declaration of Helsinki and was approved by the Ethical Committee of the Johannes Kepler University in Linz, Austria (No. K-107-16).

## 3. Statistical Analysis

Descriptive statistics are presented as medians with interquartile ranges (IQR). The data distribution was assessed by Shapiro–Wilk test. For the not normally distributed data nonparametric tests were applied. The areas under the curve (AUC) of exhaled hydrogen (H_2_) and blood glucose (BG) during lactose breath tests were calculated and compared with Kruskal–Wallis test and pairwise comparisons. The Chi-square test and the Fishers exact test were applied for symptom evaluation. The level of significance was set to 5%. The Bonferroni correction was used for multiple testing.

Statistical analyses were performed with IBM SPSS statistics version 25.0 (IBM, Armonk, NY, USA), and GraphPad Pism version 8.4.0. (GraphPad Software, San Diego, CA, USA) was used for the generation of figures.

## 4. Results

During retrospective evaluation of 279 lactose-intolerant patients, male/female 101/178, median age 40 years, age range 18–86 (IQR 25) we noticed incremental differences in exhaled H_2_ during breath tests.

**Figure 1:**
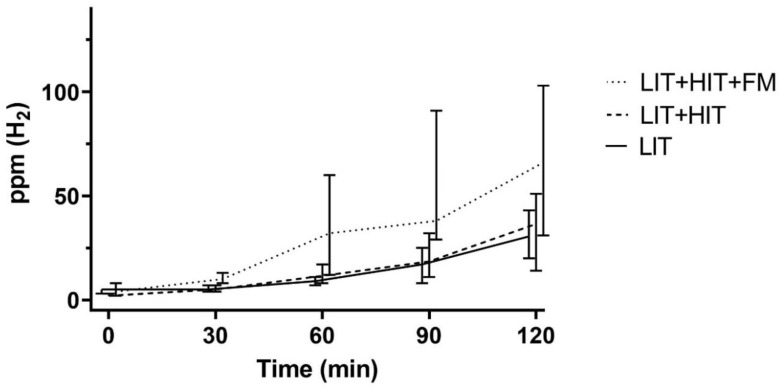
Medians and 95% confidence intervals of H_2_ ppm during lactose H_2_ breath tests in 279 patients with LIT. Included are 128 LIT-only patients, 106-LIT patients with additional HIT, and 45-LIT patients with HIT and FM. Fructose malabsorption, FM; histamine intolerance, HIT; H_2_, hydrogen; lactose intolerance, LIT; ppm, parts per million.

We separated the patients into groups and found that 128 patients—male/female 51/77, median age 39 years, age range 18–82 (IQR 24)—had LIT, only. Hydrogen breath tests did not show FM in these patients, and DAO values were >10 U/mL (median 16.9 U/mL, range 10.2–80). However, 106-LIT patients had additional HIT, male/female 36/70, median age 39 years, age range 18–78 (IQR 24). Their DAO values were <10 U/mL (median 6.4 U/mL, range 0.5–9.9). Moreover, hydrogen breath test did not show FM in this group. Forty-five patients showed LIT and HIT, combined with FM—male/female 14/31, median age 51 years, age range 18–86 (IQR 37). Accordingly, their DAO values were <10 U/mL (median 6.3 U/mL, range 1.5–9.8) and they showed >20 ppm increase of exhaled H_2_ from the baseline during a fructose malabsorption H_2_ breath test. Kruskal–Wallis test for the comparison of the AUCs of LIT and, LIT with HIT, to LIT with HIT and FM demonstrated that exhaled hydrogen values were significantly higher in patients with two-fold and triple combined food intolerance/malabsorption (*p* < 0.004 and *p* < 0.001, respectively). No significant difference was found comparing LIT to LIT and HIT (*p* = 0.55) (Figure 1).

**Figure 2:**
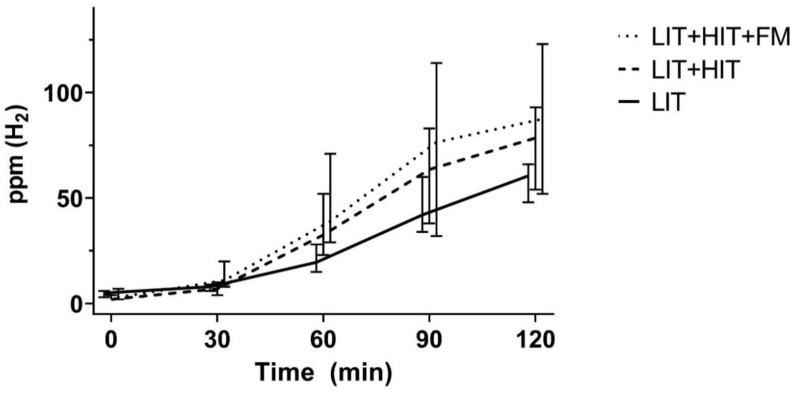
Medians and 95% confidence intervals of rising H_2_ ppm during lactose breath tests in 170 LIT patients with >20 ppm increase of expiratory H_2_ from baseline. Included are 74 LIT-only patients, 60-LIT patients with additional HIT, and 36-LIT patients with HIT and FM. Fructose malabsorption, FM; histamine intolerance, HIT; H2, hydrogen; lactose intolerance, LIT; ppm, parts per million.

Within 170 LIT patients with >20 ppm increase of expiratory H_2_ from the baseline 74 were LIT-only patients: male/female 29/45, median age 46 years, age range 19–82 (IQR 25). Hydrogen breath tests did not show FM in these patients and DAO values were >10 U/mL (median 17.5 U/mL, range 10.4–65.7). Additionally, 60 LIT patients also had HIT—male/female 21/39, median age 40.5 years, age range 18–78 (IQR 23)—with DAO values <10 U/mL (median 6.9 U/mL, range 1.5–9.9). Another, 36 LIT patients—male/female 11/25, median age 57 years, age range 18–86 (IQR 38)—had DAO values <10 U/mL (median 6.3 U/mL, range 1.5–9.8), and showed FM with >20 ppm increase of exhaled H_2_ from the baseline during the fructose H_2_ breath test. Using the Kruskal–Wallis test, the AUCs demonstrated a significant difference between all three groups (*p* = 0.024). The pairwise comparison revealed that the AUC was significantly higher in patients diagnosed with LIT, HIT, and FM, compared to patients with LIT only (*p* = 0.03). Comparing LIT to LIT combined with HIT (*p* = 0.20) and LIT with HIT to LIT, HIT and FM (*p* = 0.98) no significant differences were found (Figure 2).

According to the modality of diagnosis with H_2_ breath test results and measurements of blood glucose (BG) we found a LIT patients, diagnosed with only increasing expiratory H_2_ values from the baseline >20 ppm, in 49 patients (group A: male/female 21/28, median age 49 (IQR 33) years, age range 18–86). Their BG values increased >20 mg/dL from fasting BG. With an increase >20 ppm of expiratory H_2_ from the baseline and BG increase of <20 mg/dL we included 121 patients in the study (group B: male/female 40/81, median age 45 (IQR 24) years, age range 18–82). With BG <20 mg/dL increase we found 109 patients (group C: male/female 40/69, median age 37 (IQR 23) years, age range 18–79). Presence of serum DAO values <10 U/mL, indicative of HIT, did not influence symptoms during H_2_ breath tests.

**Figure 3:**
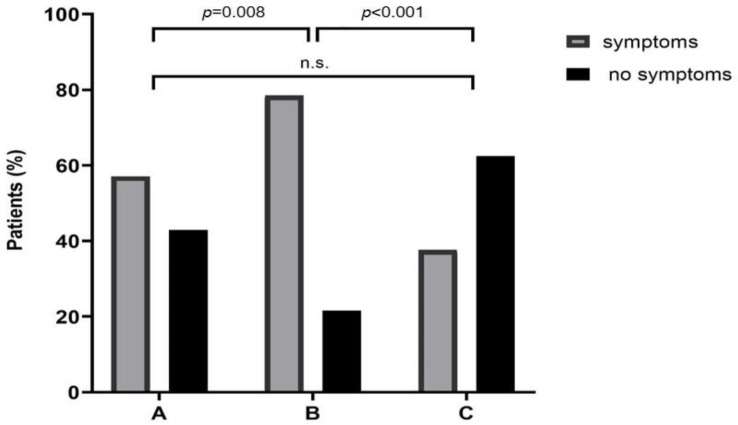
A total of 164 of 279 LIT patients indicated symptoms during lactose H_2_ breath tests. In group A (increasing expiratory H_2_ values from baseline >20 ppm) 28 patients of 49 (57%), in group B (increase >20 ppm of expiratory H_2_ from baseline and BG increase of <20 mg/dL) 95 patients of 121 (79%), and in group C (BG increase <20 mg/dL) 41 patients of 109 (38%) patients designated symptoms. BG, blood glucose; H_2_, hydrogen; LIT, lactose intolerance; ppm, parts per million; n.s., not significant.

Patients specified their GI and extra-intestinal symptoms, during breath tests on paper. GI symptoms were abdominal pain, bloating, cramps, diarrhea, vomiting, belching, and stomach rumbling. Extra-intestinal symptoms included headache, fatigue, itchy skin, and a swollen eyelid. Out of 279 LIT patients, 164 patients (59%) indicated symptoms during H_2_ breath tests. In group A: 28 of 49 (57%), group B: 95 of 121 (79%), and group C: 41 of 109 (38%) named symptoms. Significant differences were found in comparing the percentage of patients who indicated symptoms, group A to B (*p* = 0.008) and, B to C (*p* < 0.001), which are shown in Figure 3.

GI-symptoms were indicated by 160 of 279 LIT patients (57%) during H_2_ breath tests. In group A: 27 of 49 (55%), in group B: 94 of 121 (78%), and in group C: 39 of 109 (36%) showed GI symptoms only. Diabetic patients were not included and in 279 patients no significant differences in BG values (*p* = 0.301) were found. None of the patients included showed signs of *Helicobacter pylori* infection or antibodies against tissue transglutaminase. All patients >50 years old were screened by colonoscopy and no pathology was present. None of the patients showed signs of infection.

## 5. Discussion

Functional, nonspecific, non-allergic GI complaints (FNNGIC) and GI disorders, including irritable bowel syndrome (IBS) and IBS-like syndromes are one of the main reasons for consultations in primary care. From a clinical perspective there is a considerable overlap between food intolerance/malabsorption and symptom-based IBS-like gastrointestinal (GI) disorders. Currently, IBS gets classified as a functional gastrointestinal disorder, according to Rome IV criteria, but new disease models are proposed continuously [6]. However, IBS-like syndromes have symptoms resembling those of food intolerance/malabsorption. Generally, lactose is among the main triggers causing FNNGIC [7].

Food intolerance/malabsorption syndromes may cause FNNGIC and extra-intestinal symptoms, and appear in up to 20% of populations. Chronic unexplained GI complaints, as caused by IBS and IBS-like syndromes, are only symptom-based conditions. They are associated with high symptom burden and a reduced quality of life. However, IBS patients appear to believe that food, including histamine-containing food, causes their GI symptoms [8]. Usually, dietary adaptations, including limited consumption of symptom-triggering food, following individual diagnostic evaluations in food intolerance/malabsorption, demonstrate clinical benefits.

Overall, endoscopy with biopsies and histological evaluation of GI mucosa, and radiology, including ultrasound, are not exchangeable methods for the exclusion of other organic diseases, especially aged >50 years. Nonetheless, for an evaluation of patients with FNNGIC it is necessary to include LIT and fructose malabsorption H_2_ breath tests, serum diamine oxidase (DAO) determination, screening for celiac disease, and search for *Helicobacter pylori* infection [9]. LIT and FM appear, when the sugars pass through the intestines, without being digested [7] or without being absorbed [10]. In intolerance/malabsorption caused by these sugars, lactose and fructose reach the microbiota, where they act as bacterial substrate. This results in fermentation with hydrogen production. Therefore, the clinical diagnosis of LIT and FM is predominantly performed with H_2_ breath tests [11]. An impaired degradation of ingested histamine due to an anticipated gastrointestinal DAO deficiency, causes a disturbed gut flora [12]. Although, serum DAO values do not reflect gastrointestinal DAO activity [13], we demonstrated that a strict histamine-reduced diet increases serum DAO [14]. Nevertheless, the determinations of serum DAO with currently available assays have limitations, mainly due to the limited availability of enzyme-linked immunosorbent assays (ELISA) using human DAO as a standard [15]. Thus, the diagnosis of HIT may currently be supported with serum DAO measurements [16].

Only few studies have reported on combined occurrence of LIT with FM. Approximately 30% of patients with FNNGIC and carbohydrate intolerance/malabsorption may have a combination of these [4,17]. A parallel occurrence, serum DAO values <10 U/mL in more than 50% combined LIT and FM patients, indicative of additional HIT, was described [18].

Subjective perception of food intolerance/malabsorption does not always indicate LIT [19] or FM [20,21]. Investigations of varying results in lactose H_2_ breath tests led to the suspicion of various LIT phenotypes, for example, relating lactase persistence to lactose intolerance [22]. Low serum DAO values were reported with high end-expiratory H_2_, and this was described as another LIT phenotype [23]. According to diagnostic LIT groups, as described above, we found significantly more LIT patients indicating symptoms with an increase in H_2_ >20 ppm and BG <20 mg/dL (Figure 3). This suggests mucosal and metabolic differences caused by varying abilities to digest and/or absorb lactose. Moreover, this offers a possibility of different phenotypes in LIT. With high end-expiratory H_2_ values during lactose breath tests, the probability of an additional food intolerance/malabsorption being present significantly increases (Figure 2). This demonstrates an incremental influence of additional food intolerance/malabsorption on digestion.

Generally, symptoms depend on the patients’ subjective interpretation and are an abnormal experience. Great variability is also represented in different languages, because diverse terminologies for almost the identical, or almost identical, symptoms, without appropriate translations, are used [24]. In FM and LIT, the relation to symptoms in functional GI disorders remains unclear [17,20]. A variety of symptoms and combination of symptoms, GI and extra-intestinal, may appear and may be influenced by additional HIT. So far, there are few studies exploring HIT and FNNGIC [25]. Concerning another recently emerging FNNGIC disorder, namely non-celiac gluten sensitivity (NCGS), which refers to people without celiac disease avoiding gluten, we suggested a striking similarity of symptoms to HIT [26]. Withdrawal of wheat products reduces the symptom severity and improves the quality of life in persons with NCGS. Gluten-containing bakery products have also histamine and/or are consumed with histamine-containing flavorings. However, withdrawal of gluten-containing wheat products simultaneously reduces consumed histamine, which hence reduces HIT-associated symptoms. We speculate, that HIT has a far higher prevalence as the currently estimated 3% in populations [27]. If so, HIT may represent an explanation for the widespread popularity of gluten-free diets.

The interest in HIT is increasing and, further investigations certainly are needed. We present a single center experience, and suggest, that HIT offers a diagnosis which helps patients to put FNNGIC into context. So far, only few evaluations on therapeutic measures including histamine-reduced diet and supplementation with oral DAO, were reported to reduce symptoms, including FNNGIC, in patients with HIT [28,29,30,31].

We recommend, that each single patient with FNNGIC needs possible triggers of food intolerance/malabsorption, including HIT, evaluated. If detected, a registered and experienced dietician is essential to subsequently design an individually tailored diet. Each patient’s specific tolerance level needs consideration when identifying dietary restrictions to achieve long-term symptom reduction. In conclusion, we show, that in patients with LIT, the presence of additional food intolerance/malabsorption, including FM and HIT, significantly increases expiratory H_2_ values. Moreover, we demonstrate an indication that suggests HIT to embody its own, independent GI disorder as food intolerance/malabsorption.

## 6. Conclusions

In conclusion, we show, that in patients with LIT, the presence of additional food intolerance/malabsorption, including FM and HIT, significantly increases expiratory H2 values. Moreover, we demonstrate an indication that suggests HIT to embody its own, independent GI disorder as food intolerance/malabsorption.

## Abbreviations

AUC	area under the curve
BG	blood glucose
DAO	diamine oxidase
FM	fructose malabsorption
FNNGIC	functional nonspecific non-allergic gastrointestinal complaints
GI	gastrointestinal
HIT	histamine intolerance
IBS	irritable bowel syndrome
LIT	lactose intolerance
ppm	parts per million
NCGS	non-celiac gluten sensitivity
